# A novel long noncoding RNA AK029592 contributes to thermogenic adipocyte differentiation

**DOI:** 10.1093/stcltm/szae056

**Published:** 2024-08-08

**Authors:** Pengyu Hong, Dianri Wang, Yue Wu, Qi Zhang, Pan Liu, Jian Pan, Mei Yu, Weidong Tian

**Affiliations:** State Key Laboratory of Oral Diseases & National Clinical Research Center for Oral Diseases & Engineering Research Center of Oral Translational Medicine, Ministry of Education & National Engineering Laboratory for Oral Regenerative Medicine, West China Hospital of Stomatology, Sichuan University, Chengdu 610041, People’s Republic of China; Department of Oral and Maxillofacial Surgery, West China Hospital of Stomatology, Sichuan University, Chengdu 610041, People’s Republic of China; Department of Head and Neck Surgery, Sichuan Cancer Hospital & Institute, Sichuan Cancer Center, School of Medicine, University of Electronic Science and Technology of China, Chengdu 610041, People’s Republic of China; State Key Laboratory of Oral Diseases & National Clinical Research Center for Oral Diseases & Engineering Research Center of Oral Translational Medicine, Ministry of Education & National Engineering Laboratory for Oral Regenerative Medicine, West China Hospital of Stomatology, Sichuan University, Chengdu 610041, People’s Republic of China; Department of Oral and Maxillofacial Surgery, West China Hospital of Stomatology, Sichuan University, Chengdu 610041, People’s Republic of China; State Key Laboratory of Oral Diseases & National Clinical Research Center for Oral Diseases & Engineering Research Center of Oral Translational Medicine, Ministry of Education & National Engineering Laboratory for Oral Regenerative Medicine, West China Hospital of Stomatology, Sichuan University, Chengdu 610041, People’s Republic of China; Department of Oral and Maxillofacial Surgery, West China Hospital of Stomatology, Sichuan University, Chengdu 610041, People’s Republic of China; State Key Laboratory of Oral Diseases & National Clinical Research Center for Oral Diseases & Engineering Research Center of Oral Translational Medicine, Ministry of Education & National Engineering Laboratory for Oral Regenerative Medicine, West China Hospital of Stomatology, Sichuan University, Chengdu 610041, People’s Republic of China; Department of Oral and Maxillofacial Surgery, West China Hospital of Stomatology, Sichuan University, Chengdu 610041, People’s Republic of China; State Key Laboratory of Oral Diseases & National Clinical Research Center for Oral Diseases & Engineering Research Center of Oral Translational Medicine, Ministry of Education & National Engineering Laboratory for Oral Regenerative Medicine, West China Hospital of Stomatology, Sichuan University, Chengdu 610041, People’s Republic of China; Department of Oral and Maxillofacial Surgery, West China Hospital of Stomatology, Sichuan University, Chengdu 610041, People’s Republic of China; State Key Laboratory of Oral Diseases & National Clinical Research Center for Oral Diseases & Engineering Research Center of Oral Translational Medicine, Ministry of Education & National Engineering Laboratory for Oral Regenerative Medicine, West China Hospital of Stomatology, Sichuan University, Chengdu 610041, People’s Republic of China; State Key Laboratory of Oral Diseases & National Clinical Research Center for Oral Diseases & Engineering Research Center of Oral Translational Medicine, Ministry of Education & National Engineering Laboratory for Oral Regenerative Medicine, West China Hospital of Stomatology, Sichuan University, Chengdu 610041, People’s Republic of China; Department of Oral and Maxillofacial Surgery, West China Hospital of Stomatology, Sichuan University, Chengdu 610041, People’s Republic of China

**Keywords:** obesity, LncRNA AK029592, thermogenic adipocyte differentiation, adipose tissue, adipose-derived stem cells

## Abstract

Exploration of factors originating from brown adipose tissue that govern the thermogenic adipocyte differentiation is imperative for comprehending the regulatory framework underlying brown fat biogenesis and for devising therapeutic approaches for metabolic disorders associated with obesity. Prior evidence has illuminated the pivotal role of long noncoding RNAs (lncRNAs) in orchestrating thermogenesis within adipose tissue. Here, we aimed to explore and identify the critical lncRNA that could promote thermogenic adipocyte differentiation and to provide a novel strategy to treat obesity-related metabolic diseases in the future. In this study, through amalgamation with our previous lncRNA microarray data from small extracellular vesicles derived from BAT (sEV-BAT), we have identified sEV-BAT-enriched lncRNA AK029592 as a critical constituent of the thermogenic program, which actively fostered beige adipocyte differentiation and enhanced the thermogenic capacities of adipose tissue. Moreover, lncRNA AK029592 could sponge miR-199a-5p in adipocytes to stimulate thermogenic gene expression. Consequently, we concluded lncRNA AK029592 as a crucial lncRNA component of the thermogenic program that regulated beige adipocyte differentiation and white adipose tissue browning, thereby providing a novel therapeutic target and strategy in combating obesity and related metabolic diseases.

## Graphical Abstract

**Graphical Abstract:**
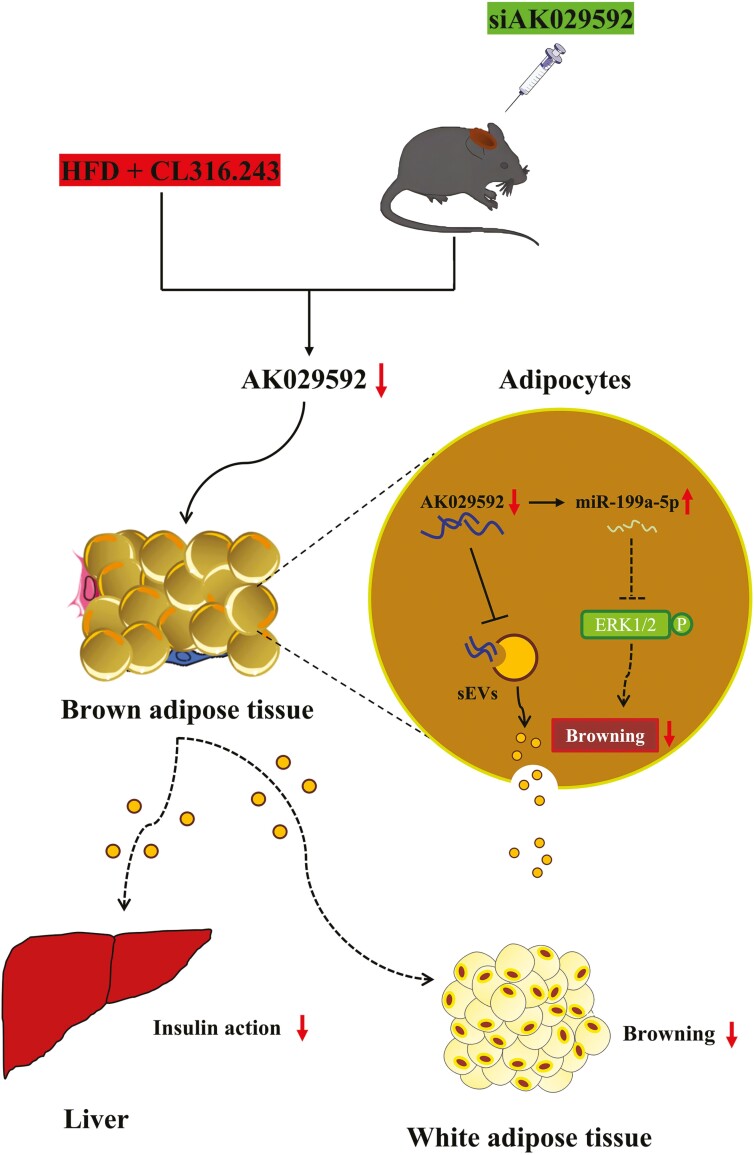

**Significance statement**

Overexpression of lncRNA AK029592 promotes thermogenic adipocyte differentiation in ASCs, while knockdown of lncRNA AK029592 impairs browning process. Knocking down the expression of lncRNA AK029592 in brown adipose tissue affected body weight, adipose tissue thermogenesis, and glucose homeostasis in CL316,243-treated high-fat fed mice. LncRNA AK029592 regulates thermogenic adipocyte differentiation process through sponging miR-199a-5p in adipocytes.

## Introduction

The global prevalence of obesity-related metabolic syndrome has surged to epidemic levels, including type 2 diabetes, cardiovascular disease, and nonalcoholic fatty liver disease^.1,2^ White adipose tissue (WAT) serves as a specialized entity for maintaining energy homeostasis, facilitating endocrine signaling, and orchestrating metabolic-immune interplay. Conversely, brown adipose tissue (BAT) is characterized by abundant multilocular lipid droplets and mitochondria, functioning to disperse glucose and fatty acids for the generation of heat.^3^ Recent studies have demonstrated that the activation of BAT thermogenesis acts as a defense mechanism against cold exposure, leading to heightened caloric expenditure, diminished adiposity, and ameliorated diabetes.^4^ While brown adipocytes significantly contribute to energy expenditure, the quantity of BAT remains relatively low and diminishes in adult humans, and this reduction in BAT correlates inversely with BMI and obesity.^5,6^ Notably, specific physiological and medicinal stimuli, such as prolonged exposure to cold and targeted activation using a selective β3-adrenergic agonist like CL-316,243, have the capacity to transform white adipocytes into mitochondria-rich, thermogenic adipocytes akin to BAT, generally termed as “beige” adipocytes.^7^ These processes are defined as WAT browning. Consequently, the activation of BAT and the induction of WAT browning represent promising approaches in combating obesity and its related metabolic disorders.^8^

A defining characteristic of brown/beige adipocyte differentiation lies in the transcriptional activation of gene programs that facilitate mitochondrial fuel oxidation and thermogenesis.^9^ Several key transcription factors, such as peroxisome proliferator-activated receptor γ (*Pparγ*), Pparα, PR domain containing 16 (*Prdm16*), and Pparγ coactivator 1α *(Pgc-1α*), have been elucidated as regulators involved in the development of brown fat.^10^ Furthermore, a number of long noncoding RNAs (LncRNAs) have been identified to regulate brown/beige adipocyte determination, differentiation, and uncoupling, including lncBlnc1, lncdPrdm16, and lncBATE1.^11-13^ LncRNAs represent a distinctive class of transcripts exceeding 200 nucleotides in length, lacking a functional open reading frame.^14^ The expression pattern of lncRNAs demonstrates notable tissue specificity and responsiveness to developmental and physiological cues.^14^ Multiple studies have underscored the regulatory role of lncRNAs in gene expression, transcriptional control, and post-transcriptional processing.^15,16^ Particularly noteworthy is their ability to act as competitive endogenous RNAs (ceRNAs), modulating mRNA expression by sponging miRNAs.^17^ Consequently, studies have delved into the functional roles and underlying mechanisms of novel batokine-like lncRNAs in the process of thermogenic adipocyte differentiation.

Recently, small extracellular vesicles (sEVs) have emerged as a novel intercellular communication system facilitating the transport of bioactive RNAs or proteins between adipose tissue and distal organs.^18^ Studies have also reported that BAT-derived sEVs (sEV-BAT) promoted energy expenditure by regulating thermogenic-related genes and oxygen consumption in recipient cells.^19,20^ Moreover, our previous studies have demonstrated that lncRNA AK029592 was enriched in sEV-BAT, brown adipocytes, and BAT, suggesting its potential association with beige adipocyte differentiation.^21^ Notably, lncRNA AK029592, a 2 kb intergenic lncRNA, appears to have ties to the insulin signaling pathway, as indicated by KEGG pathway analysis.^21^ However, the functions of lncRNA AK029592 remain largely unknown. Despite its abundant expression in BAT, it remains unclear whether lncRNA AK029592 plays a role in thermogenic adipose development or functions as a putative batokine.

In this study, we have identified lncRNA AK029592 as an inducible lncRNA in regulating thermogenic adipocyte differentiation and CL316,243-treated high-fat diet (HFD) obesity. Mechanistically, lncRNA AK029592 interacts with miR-199a-5p, thereby facilitating the activation of the thermogenic gene/protein program. Our results might implicate a prospective anti-obesity strategy by combining sEVs and lncRNA via an engineering approach in the future.

## Materials and methods

### Animals

C57BL/6 wild type male mice of 6-8-week-old were purchased from Dashuo Experimental Animal Co., Ltd.. The animal experimental protocols were approved by the Ethical Committees of the State Key Laboratory of Oral Diseases, West China School of Stomatology, Sichuan University, China (Approval number, WCHSIRB-D-2021–251). The mice were numbered and randomly assigned to either the control or the experimental group, and they were separately maintained in an individually ventilated cage system with free access to food and drinking water (5 per cage). For acute cold exposure, mice were individually caged with food withdrawn and water provided, placed in a 4°C cold room for 2 days.

### Cell isolation and culture

Adipose-derived stem cells (ASCs) were isolated and cultured as described in our previous methods.^21,22^ Briefly, BAT was collected and washed with sterile phosphate-buffered saline (PBS). Then BAT was minced into small pieces and digested with 0.075% type I collagenase for 40 minutes at 37°C. The digestions were terminated with α-minimal essential medium (α-MEM, HyClone) containing 10% fetal bovine serum (FBS, Gibco), and filtered through 40 μm filters to remove undigested tissues. The mixtures were then centrifuged, resuspended, and then maintained at 37°C with 5% CO_2_. The medium was replaced every 2 days, and the cells were passaged when they reached approximately 90% confluence.

### Adipocyte differentiation

To initiate differentiation, ASCs were plated in 6-well plates at a density of 2 × 10^5^ cells/well, cultured for 24 hours, then the cells were exposed to adipogenic induction medium for 4 days, which contained DMEM, 10% FBS, 1 μM dexamethasone, 20 nM insulin, 125 μM indomethacin, and 500 μM 3-isobutyl-1methylxanthine (IBMX). Then the cells were treated with browning induction medium supplemented with 2 µM rosiglitazone and 1 nM T3 after 4 days. Cells were switched to differentiation medium (DMEM, 10% FBS, 20 nM insulin, 2 µM rosiglitazone, and 1 nM T3) after 2 days. The cells at different time points were collected for further analysis.

### In vitro transcription/translation assay

In vitro transcription/translation assay was performed using a kit from Promega (L1171, Promega) and Transcend tRNA (L5061, Promega). Briefly, expression plasmids for luciferase and lncRNA AK029592 were mixed with a Coupled Reticulocyte Lysate System and tRNA. After incubating at 30°C for 60 minutes, translated products were separated on 10% gradient SDS polyacrylamide gel and transferred to PVDF membrane, incubated with HRP-Streptavidin (A0305, Beyotime) for X-ray autoradiography.

### siRNA and plasmid transfection

ASCs (5 × 10^4^ per well) were seeded in 24-well plates and cultured overnight. The cells were transfected with 50 nM AK029592-targeting siRNAs (Hanbio) using the Lipofectamine 2000 transfection reagent (Life Technologies) or 0.6 µg plasmid using the LipoFiter 3.0 transfection reagent (Hanbio) following the manufacturer’s instructions, respectively. The control group was treated with non-targeting siRNA or blank vector plasmids. Cells transfected with green fluorescent protein (GFP)-labeled plasmids were plated into 6-well plates and culture for 48 hours. The fluorescence images of GFP expressed by the cells were captured using a fluorescence microscope. Cells were trypsinized after the PBS wash, quenched using 2% FBS in PBS, and then used for flow cytometry.

For In situ siAK029592 injection study, 15 nmol cholesterol-modified siAK029592 or negative control (siAK029592-NC) (Ribobio) dissolved in diluted water were directly injected into the BAT of 6-week-old male C57BL/6 mice (*n* = 6 per group) every 2 days. β3-adrenergic agonist CL316,243 (Sigma) was intraperitoneally injected into mice every 2 days at 1 mg/kg body weight (Sigma). As for in vivo biodistribution, the mice were subcutaneously injected with Cy5-labeled siRNA (5 nmol/kg). The in vivo imaging system (IVIS Spectrum) was used to test fluorescence images of mice after 0, 24, 48 hours. Bodyweight and food intake were monitored. After 6 weeks of injection, glucose tolerance and insulin sensitivity were determined, and liver tissue, BAT, and subcutaneous white adipose tissue (scWAT) were collected for further analysis.

### Mito tracker staining

ASCs (1 × 10^4^) were seeded into confocal dish, transfected with 50 nM non-targeting siRNA or AK029592-targeting siRNA and induced browning as previously described. Cells were then incubated with 200 nM Mito Tracker (#9082, Cell Signaling Technology) for 30 minutes at 37°C, fixed with 4% (w/v) paraformaldehyde for 15 minutes, permeabilized with 0.5% (v/v) Triton X-100 for 15 minutes, blocked with 1% (w/v) bovine serum albumin for 30 minutes at 37°C and stain with DAPI (2 μg/mL) for 10 minutes. Adipose tissue was fixed with 4% paraformaldehyde and embedded in paraffin wax. Paraffin sections (5 μm thick) were dewaxed in xylene and rehydrated through graded ethanol. For mitochondria staining, sections were incubated with 250 nM Mito Tracker for 1 hour at room temperature. Mitochondrial abundance of differentiated cells and tissue sections were examined under a confocal microscope (Olympus FV1200), respectively. The fluorescence values were analyzed using ImageJ software.

### Mitochondrial DNA content and mitochondrial function

Mitochondrial DNA content was measured using qPCR analysis of total DNA isolated from browning-induced ASCs. The primers for mitochondrial DNA (mtND1 and mtCox1) and nuclear DNA (PECAM) are listed in Supplementary Table S1. The cellular mitochondrial function was measured using a Seahorse XFe24 Analyzer (Seahorse Agilent). For AK029592 overexpression studies, ASCs were transfected with either vector or AK029592 expression plasmid and induced browning, whereas for knockdown studies, either control siRNA (siAK029592-NC) or siAK029592 were transfected. After 48 hours, the plate was loaded into the Seahorse XFe24 Analyzer (Seahorse Agilent) and the mitochondrial function was measured according to the manufacturer’s instructions, followed by the injections of 1.5 μM ATP synthase inhibitor oligomycin, 0.5 μM mitochondrial uncoupler carbonyl cyanide-4 (trifluoromethoxy) phenylhydrazone (FCCP), and 0.5 μM complex I and III inhibitor rotenone/antimycin A (Rot/AA) (Seahorse Agilent, 103015-100).

### miRNA transfection assay

ASCs (5 × 10^4^ per well) were seeded in 24-well plates and cultured overnight. Transfection mix of 50 µL (Lipofectamine2000 (Invitrogen)) with miR-199a-5p mimics/inhibitors (Hanbio) were added into cells and incubated at 37°C for 6 hours. The transfection efficiency of mimics (25 nM) and inhibitors (50 nM) was confirmed by qPCR. To explore the functions of miR-199a-5p during beige adipocyte differentiation, AK029592-overexpressing ASCs were transfected with miR-199a-5p mimics or inhibitors and then were cultured with 0.5 mL browning induction medium (DMEM supplemented with 10% FBS, 1 μM dexamethasone, 20 nM insulin, 125 μM indomethacin, 2 µM rosiglitazone, 1 nM T3, and 500 μM 3-isobutyl-1methylxanthine (IBMX)) for 5 days (fresh browning induction medium was changed every 2 days). Total RNA and protein were extracted at day 5 after induction.

### Dual-luciferase reporter assay

AK029592 3ʹUTR region was amplified and cloned into firefly luciferase reporter vector (Hanbio), named as lncRNA AK029592-WT-Luc, and the mutagenesis was conducted in the binding regions of lncRNA AK029592 and miR-199a-5p to generate the mutant plasmid lncRNA AK029592-MUT-Luc. We used LipoFiter3.0 (Hanbio) to co-transfect the reporter plasmids and miR-199a-5p mimic into HEK-239 T cells. The solution was changed 6 hours after the transfection, and the cells were collected after 48 hours. The luciferase activity was determined using the Dual-Luciferase Reporter Assay Kit (Hanbio) following the manufacturer’s instructions. Normalized firefly luciferase activity was compared between different groups.

### qPCR

The nuclear and cytoplasmic components were separated using a Nuclear Extraction Kit (P0028, Beyotime). The total RNA of cells and tissue was then isolated using TRIzol reagent (Invitrogen). Then cDNA was synthesized from at least 1 μg of RNA using the HiScript II SuperMix (Vazyme Biotech Co., Ltd.). Quantitative polymerase chain reaction (qPCR) was carried out with a QuantStudio 6 Flex Real-Time PCR System (Life Technologies) using SYBR Premix ExTaq (Vazyme Biotech Co., Ltd.) and gene-specific primers. The data were analyzed using the 2^−ΔΔCT^ method, with ribosomal protein 36B4 was used as an internal control.

miRNAs from cells were extracted with miRNA isolation kit (TIANGEN) according to the manufacturer’s instructions then transcribed into cDNAs using miRcute Plus miRNA First-StrandcDNA Synthesis Kit (TIANGEN). cDNAs were amplified with miRcute miRNA qPCR Detection Kit (SYBR Green) (TIANGEN) using QuantStudio 6 Flex Real-Time PCR System (Life Technologies). U6 was used as an internal control. The primer sequences are listed in Supplementary Table S1.

### Western blot

To detect browning markers, an equal amount of protein (20 μg) was extracted from treated cells and tissue. Protein concentrations were measured using BCA Protein Assay Reagent (KeyGen BioTECH, KGP902). Samples were separated on a 10% sodium dodecyl sulfate-polyacrylamide gel electrophoresis (SDS-PAGE), transferred by electrophoresis to PVDF membranes (Millipore, Billerica), blocked in 5% milk for at least 1 hour and incubated with antigen-specific antibodies overnight at 4°C. UCP1 (ab23841, Abcam, 1:1000), PPARγ (ab272718, Abcam, 1:1000), and ACTIN (ab3280, Abcam, 1:5000) were from Abcam; PGC-1α (D162041, BBI, 1:1000) was from BBI Life Sciences; p-ERK (#4370, Cell Signaling Technology (CST), 1:1000), ERK (#4695, CST, 1:1000), and Lamin A/C (#4777, CST, 1:1000) were from CST. Primary antibodies were then labeled for at least 1 hour with horseradish peroxidase (HRP) conjugated secondary antibodies. Immunodetection was performed using High-sig ECL Western Blotting Substrate (Tanon,180–501) and was detected using a Bio-Rad Chemidoc imager. Band intensities were determined using ImageJ software and normalized to internal control ACTIN.

### Glucose consumption measurements

The cell culture medium of ASCs after browning induction was collected at different time points (0, 12, 24, 36, 48 hours). The concentration of glucose in the medium was determined using Glucose (HK) Assy Kit (GAHK20, Sigma) following the manufacturer’s instructions. The absorbance was measured at 340 nm with a spectrophotometer (Multiskan GO, Thermo Fisher Scientific).

### Glucose tolerance test and insulin tolerance test

For Glucose tolerance test (GTT), mice were fasted for 8 hours. After basal glucose measurement, glucose (2 g/kg) was injected and blood glucose was measured from the tail tip at 0, 30, 60, 90 and 120 minutes by using a glucometer. For insulin tolerance test (ITT), mice were fasted for 5 hours. Following basal glucose measurement at 0 minute time point, insulin (0.75 U/kg) was injected in every group and blood glucose from the tail tip was measured at 0, 30, 60, 90, and 120 minutes.

### Histology

The tissue samples were fixed in 10% neutral-buffered formalin for 24 hours, dehydrated with a graded alcohol series, and embedded in paraffin. Sections with a thickness of 4 μm were stained with hematoxylin and eosin (H&E). BAT and scWAT tissues were incubated for 2 hours at 60°C, deparaffinized, and rehydrated. Antigen retrieval was performed using citrate buffer (pH6) at 97°C for 20 minutes. Endogenous peroxidase activity was blocked by incubating the sections with 3% hydrogen peroxide for 10 minutes at room temperature. Nonspecific binding of the antibody was blocked by incubating the slides with 5% normal goat serum in PBS containing 0.1% Tween 20 (PBST) for 1 hour at room temperature. The slides were then incubated with primary antibodies against UCP1 (1:200, Abcam) overnight at 4°C followed by secondary antibodies (Alexa-555 conjugated anti-rabbit (1:300, CST)) for 2 hours. Cells were imaged on a confocal microscope (Olympus FV1200, Japan). Images were stacked to ensure equal adjustments to all images.

### Statistical analysis

Results were presented as mean ± SD. Significance between the 2 groups was assessed by 2 tailed Student’s *t*-test. The comparisons between multiple groups were carried out using one-way ANOVA test. A *P*-value <.05 was considered statistically significant.

## Results

### Identification of lncRNA AK029592 as an inducible lncRNA during browning induction

**Figure 1.**
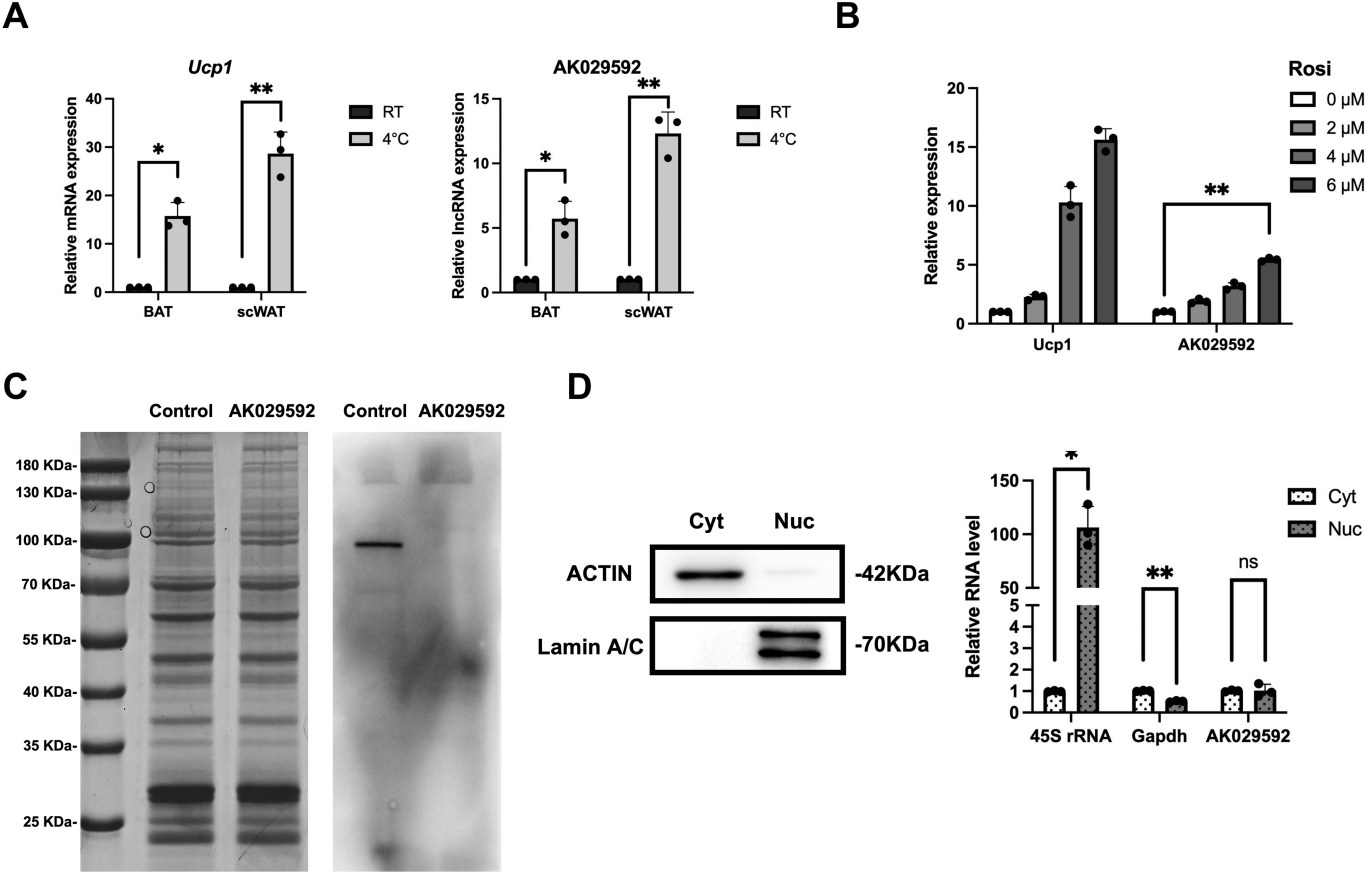
Identification of AK029592 as an inducible lncRNA in browning differentiation. (A) qPCR analysis of gene expression 2 days after treatment of room temperature and 4°C (*n* = 3). (B) qPCR analysis of gene expression during browning differentiation with different concentrations of rosiglitazone (*n* = 3). (C) In vitro transcription/translation assay using control and AK029592 constructs. Shown are Coomassie staining (left) and Transcend tRNA (right). (D) Immunoblots and qPCR analysis of cytosolic (Cyt) and nuclear (Nuc) fractions. Data were represented as mean ± SD and analyzed by Student’s *t* test, *P < .05, **P < .01.

According to our previous studies, the expression of lncRNA AK029592 was upregulated during thermogenic adipocyte differentiation in ASCs and enriched in brown adipose tissue.^21^ Moreover, we found that cold stimulation upregulated the expression of lncRNA AK079912 and *Ucp1* in both BAT and scWAT, relative to the expression levels at room temperature (Figure 1A). In addition, the expression of lncRNA AK029592 was also highly induced during browning induction, along with the increase of the concentration of browning stimuli rosiglitazone (Figure 1B). Thus, the expression analyses established that lncRNA AK029592 was involved in adipocyte browning processes.

We then explored the non-coding nature of lncRNA AK029592 through coding-potential analysis. LncRNA AK029592 had a low probability of containing a protein-coding open reading frame (Supplementary Figure S1A). We performed phylogenetic information-based codon substitution frequency (PhyloCSF) analyses for lncRNA AK029592, which was a genomic tool that distinguished protein-coding from noncoding transcripts. Note that the PhyloCSF score of lncRNA AK029592 was negative throughout the entire gene (for all 6 tracks), indicating a lack of sequence conservation, which supports its proper annotation as a noncoding RNA (Supplementary Figure S1B). Further, in vitro transcription-translation assay demonstrated that lncRNA AK029592 failed to produce any proteins and thus confirmed that lncRNA AK029592 was bona fide a lncRNA (Figure 1C).

Moreover, the subcellular locations of lncRNAs are generally exquisitely regulated, reflecting their certain biological functions and the particular developmental stage that the cell has experienced.^23^ qPCR analyses of nuclear and cytoplasmic RNA showed that lncRNA AK029592 was primarily localized in both nuclear and cytoplasmic compartments (Figure 1D). As control, ACTIN and Lamin A/C proteins were detected in the cytoplasmic and nuclear fractions, respectively. In addition, 45S ribosomal RNA was primarily localized in the nucleus, whereas *Gapdh* was mainly found in the cytoplasmic fraction (Figure 1D).

### LncRNA AK029592 promoted the thermogenic gene program in ASCs

**Figure 2.**
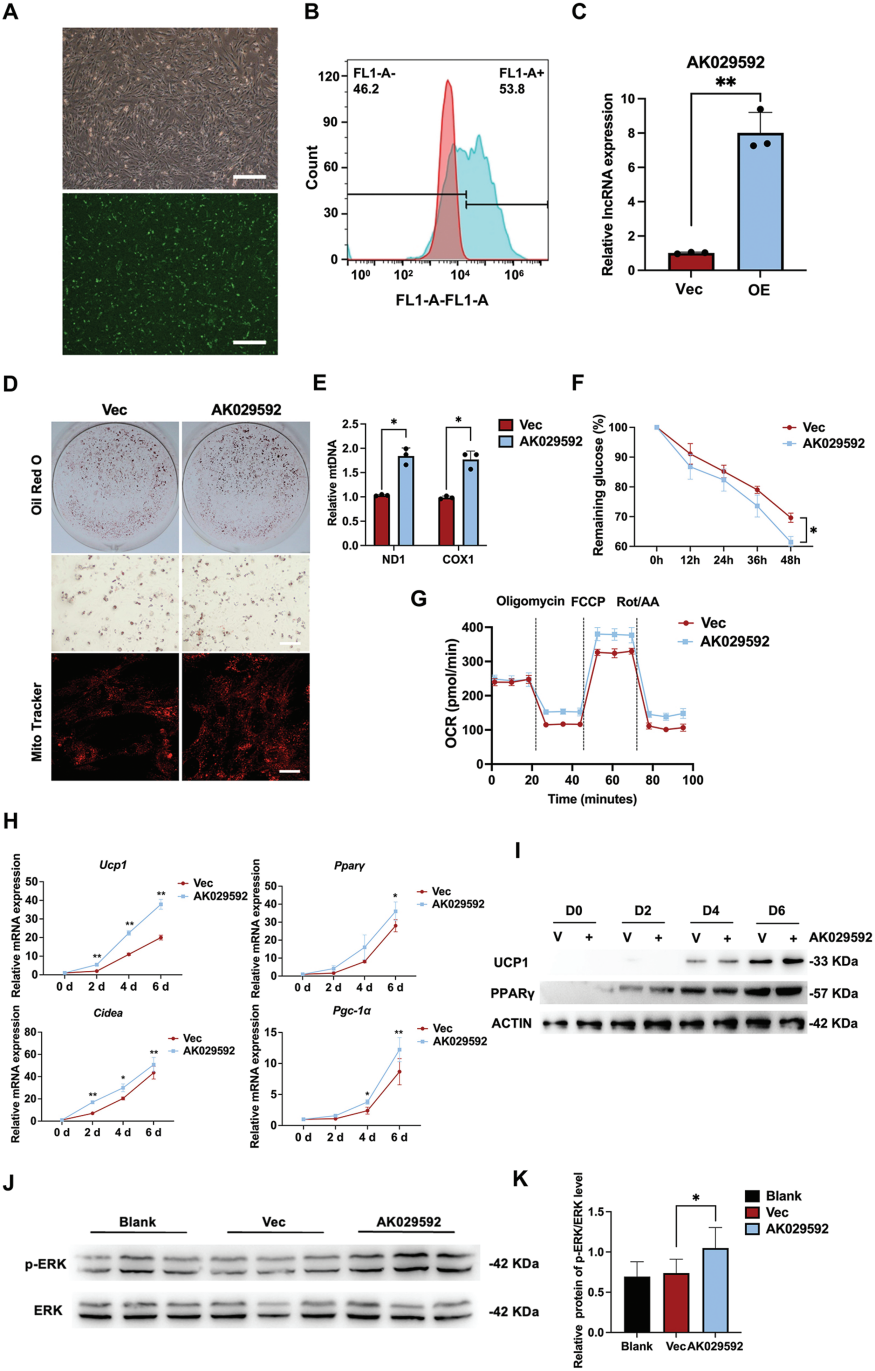
LncRNA AK029592 promoted beige adipocyte differentiation. (A) The representative microscope and GFP fluorescence images of ASCs 2 days after transfected with blank vector and lncRNA AK029592 overexpression plasmids. Scale bar = 200 μm. (B) Flow cytometry analysis of ASCs 2 days after transfected with blank vector and lncRNA AK029592 overexpression plasmids. (C) Relative expression levels of lncRNA AK029592 2 days after transfected with blank vector and lncRNA AK029592 overexpression plasmids (*n* = 3). (D) Oil Red O and Mito Tracker staining of differentiated beige adipocytes. Scale bar (Oil Red O staining) = 200 μm; Scale bar (Mito Tracker staining) = 50 μm. (E) qPCR analyses of mitochondrial DNA content in blank vector and lncRNA AK029592 overexpression adipocytes (*n* = 3). (F) Glucose consumption in beige adipocytes after transfected with lncRNA AK029592 overexpression plasmids (*n* = 3). (G) Mitochondrial oxygen consumption rate (OCR) curves of blank vector group andd lncRNA AK029592 overexpression group. (H) qPCR analysis of thermogenic related gene expression during beige adipocyte differentiation after transfected with blank vector or lncRNA AK029592 overexpression plasmids (*n* = 3). (I) Immunoblots analysis of thermogenic related protein expression levels in adipocyte lysates during browning differentiation. (J-K) Immunoblots of expression levels of phosphorylated ERK (pERK) and ERK in adipocyte lysates after browning differentiation (*n* = 3). Data were represented as mean ± SD and analyzed by student t test or one-way ANOVA, *P < .05, **P < .01, ***P < .001.

To explore the role of lncRNA AK029592 in beige adipocyte differentiation of ASCs, we transfected ASCs with a vector control or a plasmid encoding lncRNA AK029592 and monitored adipocyte gene expression at different time points following browning induction. First, we confirmed that the plasmid encoding lncRNA AK029592 could be transfected into ASCs successfully through fluorescence microscope, flow cytometry, and qPCR analysis (Figure 2A-C). Oil Red O staining indicated that overexpression of lncRNA AK029592 did not affect lipid accumulation in differentiated adipocytes (Figure 2D). In contrast, mitochondrial mass and DNA content were increased in AK029592-overexpressing adipocytes (Figure 2D, E). In addition, we found an upregulation of glucose consumption in the AK029592-overexpression group, which implied that the numbers of beige adipocytes were increased, and the browning process was enhanced (Figure 2F). Overexpressing of lncRNA AK029592 in ASCs indicated a higher oxygen consumption rate (OCR), showing increased mitochondrial uncoupling, consistent with the characteristics of beige adipocytes (Figure 2G). Moreover, qPCR analysis revealed that overexpression of lncRNA AK029592 significantly increased mRNA expression of key thermogenic markers, including *Ucp1*, *Pparγ*, *Cidea*, and *Pgc-1α* (Figure 2H). UCP1 and PPARγ protein levels were also increased in AK029592-overexpressing adipocytes during browning differentiation (Figure 2I). Studies have shown that the activation of ERK signaling was closely correlated in promoting beige adipocyte differentiation and WAT browning.^24,25^ Consistently, we found that rosiglitazone-stimulated phosphorylation of ERK in browning process was significantly promoted by overexpression of lncRNA AK029592 (Figure 2J-K). Collectively these gain-of-function studies strongly implicated lncRNA AK029592 as a lncRNA activator of brown adipogenesis.

### LncRNA AK029592 was required for beige adipocyte differentiation of ASCs

**Figure 3.**
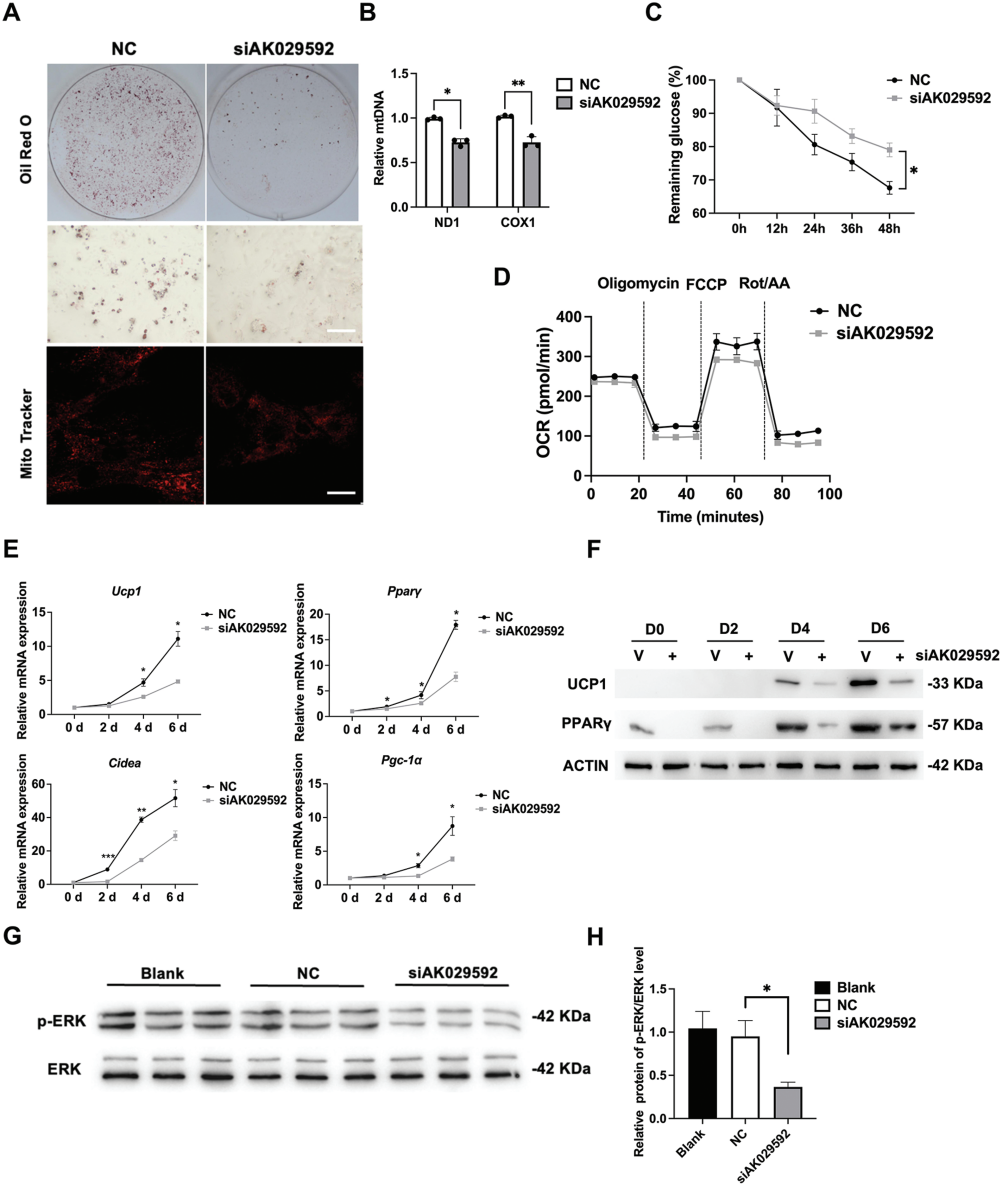
LncRNA AK029592 was required for beige adipocyte differentiation. (A) Lipid droplets and Mito Tracker staining. Scale bar (Oil red O staining) = 200 μm. Scale bar (Mito Tracker staining) = 50 μm. ASCs transfected with negative control (NC) or siRNA targeting lncRNA AK029592 were differentiated for 6 days (B) qPCR analyses of mitochondrial DNA content in siAK029592-NC and siAK029592 adipocytes (*n* = 3). (C) Glucose consumption in beige adipocytes after transfected with siAK029592 (*n* = 3). (D) Mitochondrial OCR curves of siAK029592-NC group or siAK029592 group (E) qPCR analysis of thermogenic related gene expression during beige adipocyte differentiation after transfected with siAK029592-NC and siAK029592 (*n* = 3). (F) Immunoblots of thermogenic-related protein expression levels in adipocyte lysates during browning differentiation. (G-H) Immunoblots of phosphorylated ERK (p-ERK) and ERK expression levels in adipocyte lysates after browning differentiation (*n* = 3). Data were represented as mean ± SD and analyzed by Student’s *t*-test or one-way ANOVA. *P < .05, **P < .01, ***P < .001.

We next performed RNAi knockdown studies to assess the significance of lncRNA AK029592 in beige adipocyte differentiation using siRNA targeting lncRNA AK029592. We transfected ASCs with control or siAK029592 and subjected transfected cells to browning induction for 6 days. As expected, siAK029592 expression significantly decreased lncRNA AK029592 levels in ASCs (Supplementary Figure S2). RNAi knockdown of lncRNA AK029592 severely impaired beige adipocyte differentiation, as revealed by reduced lipid accumulation, mitochondrial mass, and DNA content (Figure 3A, B). Moreover, the process of glucose consumption was impaired in the siAK029592 group (Figure 3C). The OCR was also lower in ASCs with depleted lncRNA AK029592 expression (Figure 3D). In addition, the brown fat marker gene expression was greatly decreased during browning induction process, including *Ucp1*, *Pparγ*, *Cidea*, and *Pgc-1α* (Figure 3E). Consistently, UCP1 and PPARγ protein levels were also greatly reduced by RNAi knockdown of lncRNA AK029592 (Figure 3F). Further, phosphorylation of ERK in browning process was significantly lower by uptake of siAK029592 (Figure 3G, H). These loss-of-function studies illustrated that lncRNA AK029592 was required for the activation of thermogenic gene programs in vitro.

### Knocking down lncRNA AK029592 in BAT blunted CL316.243 mediated obesity combat in HFD-fed mice

**Figure 4.**
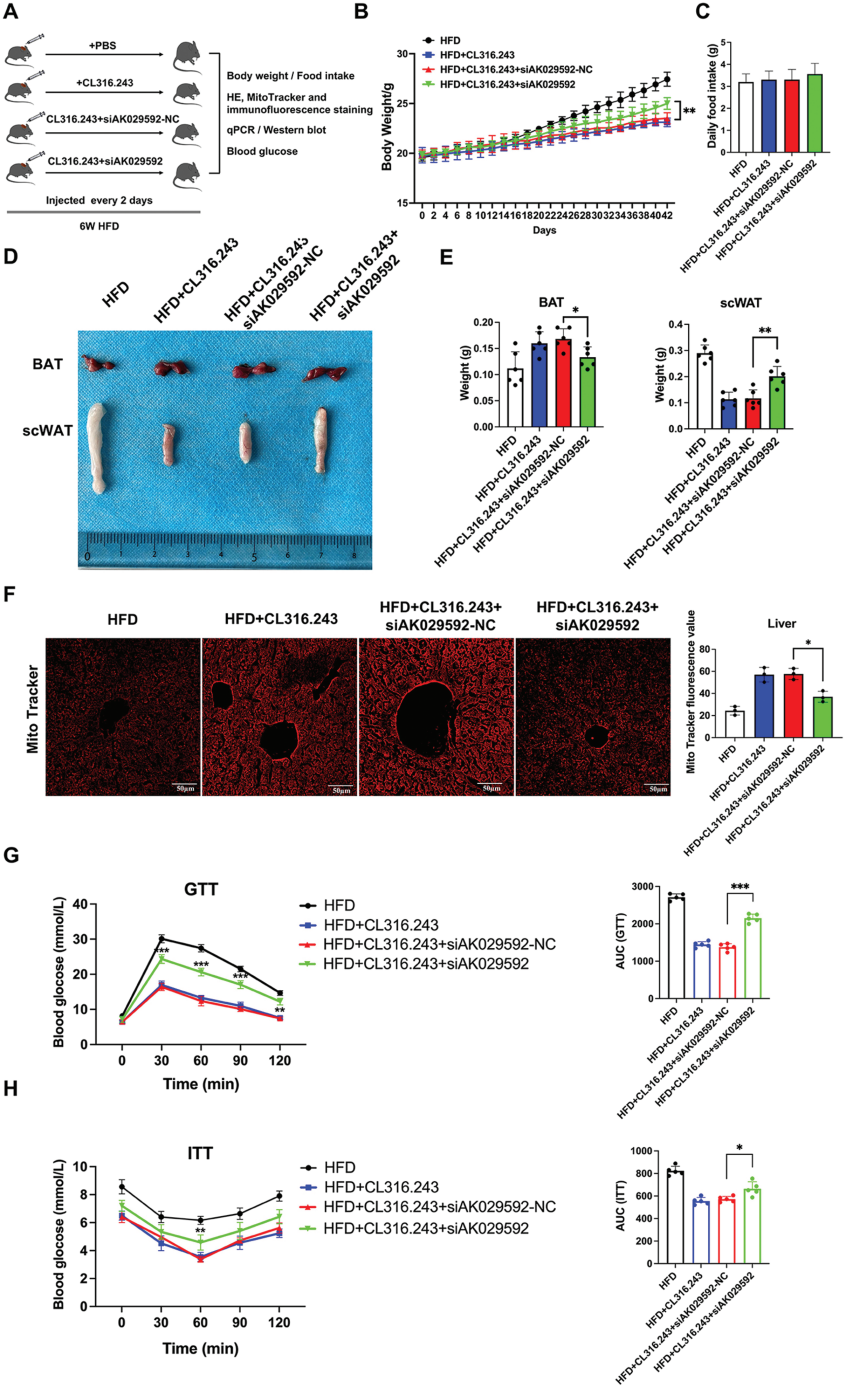
Knocking down lncRNA AK029592 in BAT blunted CL316.243 mediated obesity combat in HFD-fed mice. (A) Schematic study plan where 8 weeks old, male C57BL/6 mice were fed with HFD for 6 weeks, HFD-fed mice were directly injected with CL316,243 + siAK029592-NC and CL316,243 + siAK029592 for 6 weeks; HFD-fed mice treated with CL316,243 or PBS were used as controls. (B) Body weight change during the experiment (*n* = 6). (C) Diet intake of each group. (D-E) BAT, scWAT weight was evaluated after of intervention (*n* = 6). (F) Representative Mito Tracker images and Mito Tracker fluorescence value of liver tissue in each group. (G) GTT were evaluated after the intervention, respectively (*n* = 5). (H) ITT was performed in mice after the intervention, respectively (*n* = 5). Data were represented as mean ± SD and analyzed by one-way ANOVA. *P < .05, **P < .01, ***P < .001.

It was well-documented that CL316,243 (a β3 agonist) could induce white adipocyte populations to beige adipocytes, thus increasing energy consumption and combating obesity.^26,27^ Considering the role of lncRNA AK029592 in WAT browning, we asked whether it could play a role in CL316,243 mediated obesity combating in vivo. To explore this possibility, we firstly found that the BAT injected with siAK029592 lost bioluminescence signals after 2 days (Supplementary Figure S3). We therefore knocked down lncRNA AK029592 by locally injected siAK029592 into BAT every 2 days for 6 weeks while the mice were induced for obesity (Figure 4A). We noted that CL316,243 restricted HFD-induced weight gain while this effect was attenuated when lncRNA AK029592 was knocked down in BAT (Figure 4B). Cumulative diet intake was unaltered between the groups (Figure 4C). CL316,243 + siAK029592 injected mice significantly increased BAT and scWAT mass as compared to the HFD mice treated with CL316,243 + siAK029592-NC (Figure 4D, E). Moreover, Mito Tracker of liver tissue indicated that CL316,243 + siAK029592-NC administration attenuated HFD-induced mitochondrial mass loss while it was not obvious in the CL316,243 + siAK029592 treatment group (Figure 4F). Besides, CL316,243 + siAK029592-NC administration improved glucose tolerance and insulin sensitivity (Figure 4G, H). However, these effects were significantly attenuated when the mice were treated with CL316,243 + siAK029592. Additionally, injection of siAK029592 in BAT did not show obvious organ toxicity in heart, liver, spleen, lung, and kidney (Supplementary Figure S4). Conclusively, knocking down lncRNA AK029592 in BAT impaired CL316,243 mediated body weight loss and insulin sensitivity improvement in HFD-fed obese mice.

### Knocking down lncRNA AK029592 impaired CL316,243 mediated thermogenic related functions in BAT

**Figure 5.**
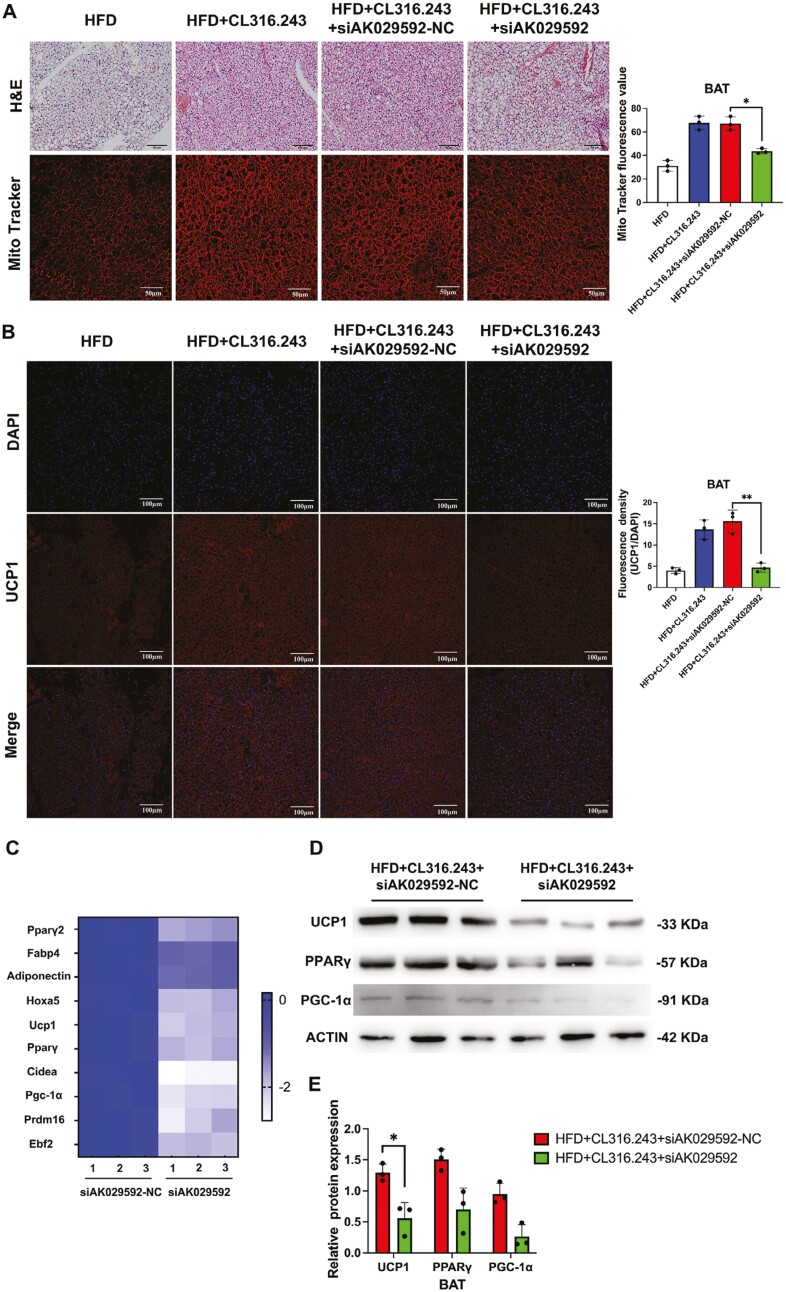
Knocking down lncRNA AK029592 impaired CL316,243 mediated thermogenic-related functions in BAT. (A) Representative H&E, Mito Tracker staining and fluorescence value in BAT in mice treated with CL316,243 + siAK029592-NC or CL316,243 + siAK029592. Scale bar (H&E staining) = 100 μm, Scale bar (Mito Tracker staining) = 50 μm (*n* = 3). (B) Representative UCP1 immunohistochemical staining and quantitative analysis of fluorescence density (UCP1/DAPI) in BAT in mice treated with CL316,243 + siAK029592-NC or CL316,243 + siAK029592. Scale bar = 100 μm (*n* = 3). (C) Heatmap of gene expression in BAT (*n* = 3). (D-E) Immunoblots of thermogenic-related proteins in BAT (*n* = 3). Data were represented as mean ± SD and analyzed by Student’s *t* test. *P < .05.

As shown above, lncRNA AK029592 played a pivotal role in the regulation of beige adipocyte differentiation. Therefore, it was reasonable to speculate that the downregulation of lncRNA AK029592 would affect the browning process of BAT. To further investigate the effects of lncRNA AK029592 on CL316,243-induced browning in vivo, siAK029592 was directly injected into BAT of C57BL/6 mice every 2 days separately for 6 weeks. qPCR analysis revealed that the expression of lncRNA AK029592 in BAT was suppressed by siAK029592 (Supplementary Figure S5). Histological and Mito Tracker studies of BAT indicated that siAK029592 administration attenuated the increase in the amount of brown adipose cells and mitochondrial mass induced by CL316,243 while they were significantly upregulated in the CL316,243 + siAK029592-NC group (Figure 5A). The amount of UCP-1 positive adipocytes in BAT were also downregulated when the mice were administrated with CL316,243 + siAK029592 while there was obviously increased when the mice were administrated with CL316,243 + siAK029592-NC (Figure 5B). Moreover, the elevated expressions of *Pgc-1α*, *Cidea*, *Prdm16*, *Ucp1* and other thermogenic-related genes in the BAT of CL316,243 + siAK029592-NC treated mice were significantly impaired by siAK029592 (Figure 5C). Further, Western blot analysis also showed that brown adipocytes injected by siAK029592 partially failed to form typical characteristics of brown fat, as indicated by relatively lower PPARγ, UCP1, and PGC-1α expression compared to siAK029592-NC (Figure 5D, E). In conclusion, these results suggested that knocking down AK029592 in BAT could impair CL316,243 mediated BAT browning.

### Knocking down lncRNA AK029592 impaired CL316,243 mediated WAT browning

**Figure 6.**
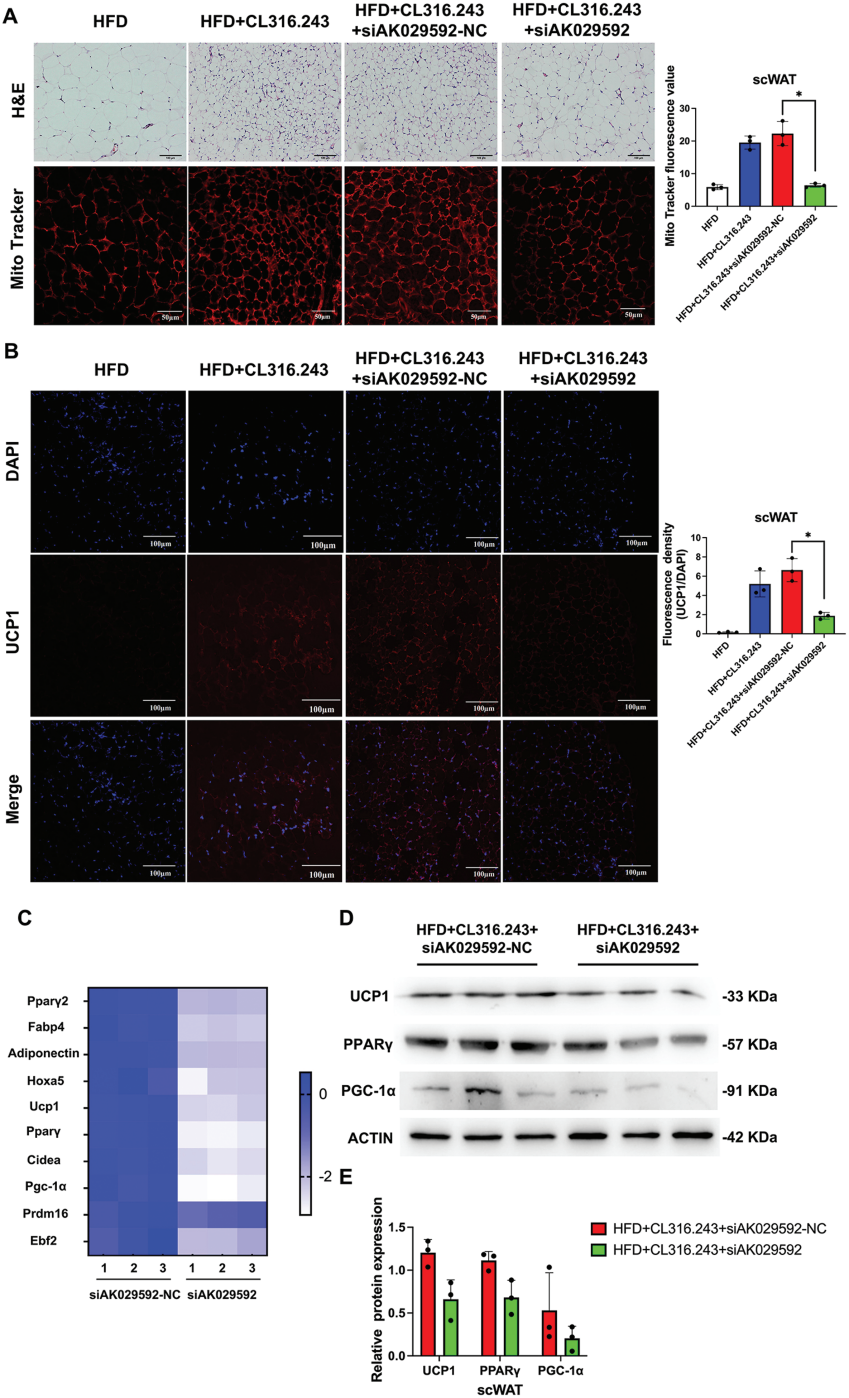
Knocking down lncRNA AK029592 impaired CL316,243 mediated scWAT browning. (A) Representative H&E, Mito Tracker staining and fluorescence value in scWAT in mice treated with CL316,243 + siAK029592-NC or CL316,243 + siAK029592. Scale bar (H&E staining) = 100 μm. Scale bar (Mito Tracker staining) = 50 μm (*n* = 3). (B) Representative UCP1 immunohistochemical staining and quantitative analysis of fluorescence density (UCP1/DAPI) in scWAT in mice treated with CL316,243 + siAK029592-NC or CL316,243 + siAK029592. Scale bar = 100 μm (*n* = 3). (C) Heatmap of gene expression in scWAT (*n* = 3). (D-E) Immunoblots of thermogenic-related proteins in scWAT (*n* = 3). Data were represented as mean ± SD and analyzed by Student’s *t*-test.

Considering lncRNA AK029592 in BAT was knocked down by local injection of siAK029592, we next continued to investigate the effects of lncRNA AK029592 on CL316,243-induced WAT browning in vivo. Surprisingly, we found that the expression of lncRNA AK029592 in scWAT was also suppressed by siAK029592 (Supplementary Figure S5). Histologically, knockdown of lncRNA AK029592 caused a substantial decrease in the number of multilocular adipocytes and mitochondrial mass within the scWAT, along with a decrease in UCP1 positive cells compared to CL316,243 + siAK029592-NC group (Figure 6A, B), which implied that knockdown of lncRNA AK029592 in BAT impaired scWAT browning. In addition, we also found that CL316,243 + siAK029592-NC promoted expressions of all thermogenic marker genes while the elevated expressions of thermogenic markers significantly decreased when lncRNA AK029592 was downregulated (Figure 6C). Consistently, PPARγ, UCP1, and PGC-1α protein levels in scWAT were also reduced by RNAi knockdown of lncRNA AK029592 (Figure 6D, E). These results demonstrated that lncRNA AK029592 was involved in the regulation of CL316,243-induced scWAT browning in vivo.

### LncRNA AK029592 functioned as an endogenous miR-199a-5p sponge in beige adipocyte differentiation

**Figure 7.**
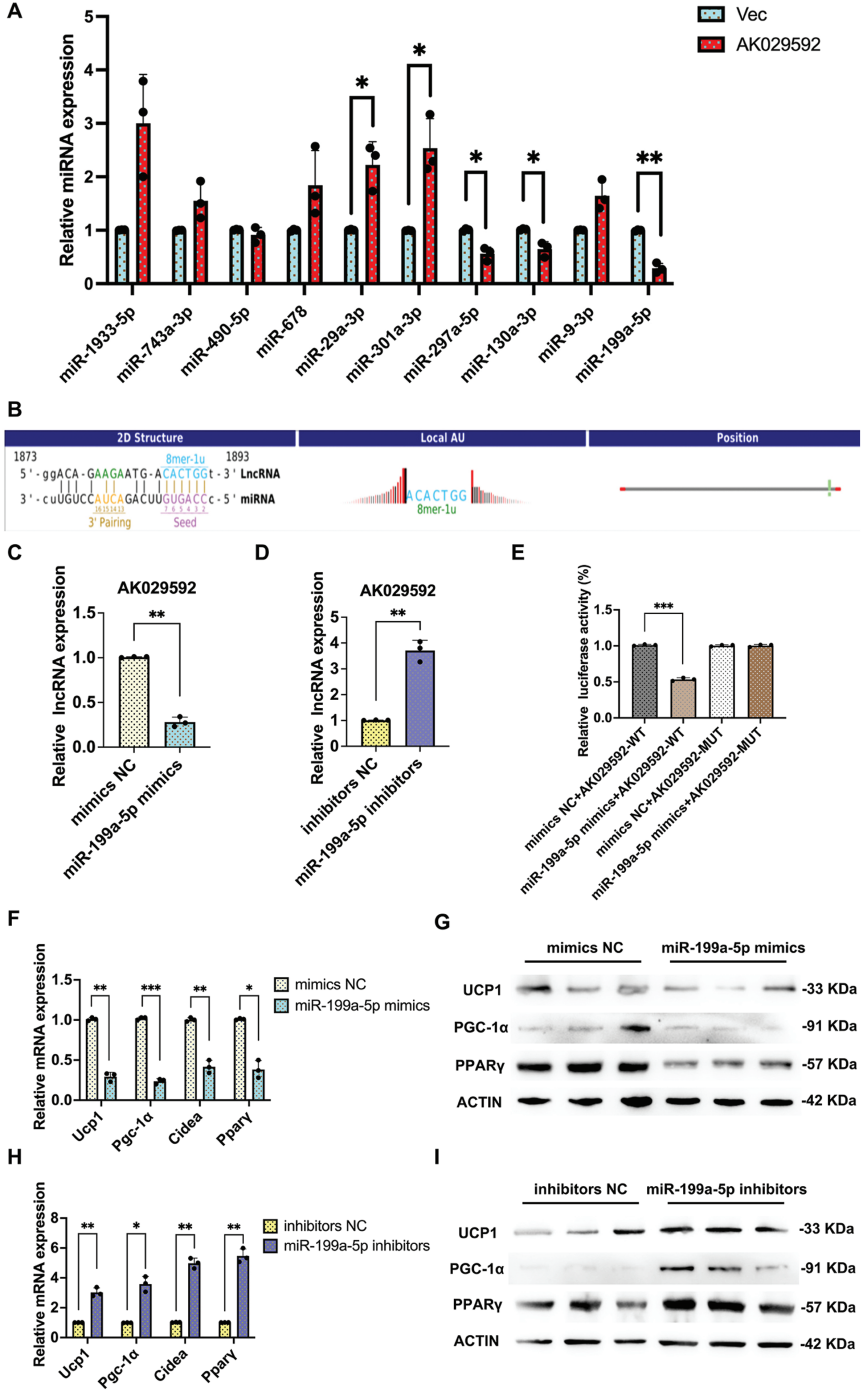
LncRNA AK029592 functioned as an endogenous miR-199a-5p sponge in browning process. (A) qPCR analysis of the expression of miR-199a-5p after overexpression of lncRNA AK029592 (*n* = 3). (B) Predicted interaction between miR-199a-5p and its putative binding sites in the 3ʹUTR of lncRNA AK029592. (C-D) qPCR analysis of the expression of lncRNA AK029592 in ASCs after transfected with miR-199a-5p mimics/inhibitors (*n* = 3). (E) Normalized luciferase activity 48 hours after co-transfection of mimic control (mimics NC) or miR-199a-5p mimics together with lncRNA AK029592-WT or lncRNA AK029592-MUT (*n* = 3). (F) qPCR analysis of thermogenuic related gene expression in lncRNA AK029592 overexpression adipocytes transfected with miR-199a-5p mimics NC/mimics (*n* = 3). (G) Immunoblots of thermogenic-related proteins in lncRNA AK029592 overexpression adipocytes transfected with miR-199a-5p mimics NC/mimics (*n* = 3). (H) qPCR analysis of thermogenic related gene expression in lncRNA AK029592 overexpression adipocytes transfected with miR-199a-5p inhibitors NC/inhibitors (*n* = 3) (I) Immunoblots of thermogenic related proteins in lncRNA AK029592 overexpression adipocytes transfected with miR-199a-5p inhibitors NC/inhibitors (*n* = 3). Data were represented as mean ± SD and analyzed by Student’s *t* test. *P < .05, **P < .01, ***P < .001.

Previous studies have investigated that lncRNA can function as miRNA sponges.^28^ To predict the potential target miRNAs of lncRNA AK029592, 10 miRNAs were selected from the overlap among the 3 databases (miRanda, LncRBase, and starBase). Among these 10 miRNAs, qPCR revealed that the miRNA expression of miR-199a-5p was significantly downregulated by overexpression of lncRNA AK029592 (Figure 7A). Importantly, studies have shown that knockdown of miR-199a-5p could increase thermogenic gene expression and mitochondrial function in beige adipocytes. miR-199a-5p was a key negative regulator of brown and beige fat development and thermogenesis.^29^ Moreover, a bioinformatic analysis by TargetScan database elucidated that lncRNA AK029592 shares miRNA response elements with miR-199a-5p (Figure 7B).

We then transfected miR-199a-5p mimics and inhibitors into ASCs, and qPCR analysis revealed that lncRNA AK029592 was greatly elevated by miR-199a-5p inhibitors while this effect was reversed by miR-199a-5p mimics (Figure 7C, D). Therefore, we mutated these miRNA response elements of AK029592 and cloned them into a luciferase reporter containing the 3ʹ-untranslated regions (3ʹ-UTR) of lncRNA AK029592. miR-199a-5p mimics were transfected into HEK-239T cells, and the luciferase activities of the lncRNA AK029592-MUT reporters were stronger than those of the wild-type (LncRNA AK029592-WT) reporter, indicating that miR-199a-5p could directly bind to lncRNA AK029592 (Figure 7E). These results confirmed that lncRNA AK029592 could serve as an endogenous miR-199a-5p sponge. To assess the thermogenic-related ability of miR-199a-5p during the beige adipocyte differentiation process, we analyzed thermogenic gene and protein expression on day 5 before and after rosiglitazone induction. As expected, lncRNA AK029592 + miR-199a-5p mimics group robustly decreased thermogenic gene (*Ucp1*, *Pparγ*, *Cidea*, and *Pgc-1α*) and protein expression (UCP1, PGC-1α, and PPARγ) compared with lncRNA AK029592-overexpressing group, while lncRNA AK029592 + miR-199a-5p inhibitor group showed the opposite (Figure 7F-I). Accordingly, these results demonstrated that lncRNA AK029592 regulated beige adipocyte differentiation by sponging miR-199a-5p.

## Discussion

Elucidating potential functional factors involved in thermogenic adipocyte differentiation might give rise to novel attractive strategies to combat obesity-associated metabolic disorders.^6^ Previous studies showed that batokines-like lncRNAs as crucial regulatory molecules influencing both BAT development and WAT browning.^11-13^ According to our previous studies, we noted a substantial enrichment of lncRNA AK029592 expression in both BAT and brown adipocytes.^21^ In the current study, we observed a significant upregulation of lncRNA AK029592 expression in response to cold stimulation and elevated concentrations of rosiglitazone. Therefore, we inferred that lncRNA AK029592 might play a crucial role in the biological processes governing thermogenic adipocyte differentiation. To further confirm our hypothesis, we transfected ASCs with AK029592 plasmids/siAK029592 to respectively elevate or reduce lncRNA AK029592 expression levels. Subsequently, we assessed the ensuing impacts during beige adipocyte differentiation. The gain and loss-of-function assays conclusively demonstrated that lncRNA AK029592 functioned as a pivotal regulator of mitochondrial function and the expression of genes/proteins involved in thermogenesis in vitro. Moreover, through a combination of bioinformatic analysis and dual-luciferase reporter assays, we found that lncRNA AK029592 could serve as an endogenous sponge for miR-199a-5p. This interaction was further supported by the modulation of thermogenic gene and protein expression, confirming the influence of miR-199a-5p in the beige adipocyte differentiation process orchestrated by lncRNA AK029592. Moreover, when lncRNA AK029592 was knocked down in BAT in vivo, the CL316,243-treated HFD mice displayed impaired glucose metabolism reduced accumulation of beige adipocytes, and a decline in thermogenic gene and protein. Accordingly, the targeting of lncRNA AK029592 might emerge as a promising therapeutic strategy to facilitate browning and counteract obesity.

Like protein regulators, many lncRNAs could serve as a novel class of regulatory molecules that exhibited remarkable tissue specificity and participate in diverse biological processes.^14^ In this study, we demonstrated that lncRNA AK029592 could alter the expressions of several thermogenic marker genes and proteins in adipocytes, especially *Ucp1*, *Pgc-1α,* and *Cidea* by sponging miR-199a-5p. Studies showed that expression of the miR-199a was associated with myogenic differentiation and myogenesis, as beige adipocytes have been indicated to originate primarily from smooth muscle cells.^30-32^ Moreover, miR-199a-5p suppressed brown adipocyte differentiation and beige fat development by directly targeting *Prdm16* and *Pgc-1α*, 2 key transcriptional regulators of adipose browning.^29^ Thus, the lncRNA AK029592-miR-199a-5p axis might act as a key regulator of brown and beige adipocyte development and function by targeting a regulatory gene cascade involved in brown and beige adipogenesis and thermogenesis.

Although we found that lncRNA AK029592 could promote browning process, the effects of which on other metabolic organs were elusive. Obesity-related metabolic disorders often involve changes in other insulin-sensitive tissues like the liver and skeletal muscle, leading to conditions such as glucose intolerance.^33,34^ In this study, we observed that the injection of siAK029592 in BAT exerted a significant effect in counteracting CL316,243-induced alterations in whole-body glucose homeostasis. This was evidenced by a reduction in mitochondrial mass in the liver, as well as the elevated glucose tolerance and insulin sensitivity. However, further study is still needed to elucidate the precise mechanism of lncRNA AK029592 confers metabolic benefits on other metabolic tissues.

Numerous studies have explored enhancing the thermogenic functions of BAT to combat obesity-related metabolic syndrome, targeting various pathways such as G protein-coupled receptors, transient receptor potential channels, nuclear receptors, and other related pathways.^8^ BAT contributes to whole-body fuel and energy metabolism through the thermogenesis-dependent role of brown adipocytes and releases specific secreted factors, notably sEV-BAT, which play a crucial role in this metabolic orchestration.^20,35^ Our previous studies found that lncRNA AK029592 was highly expressed in sEV-BAT, which could stimulate beige adipocyte differentiation and significantly impact HFD-induced obesity and whole-body glucose homeostasis.^20,21^ These effects were attributed to increased energy expenditure and the reduction of adipose tissue inflammation.^20,21^ Given that sEVs are entirely cell-free and exhibit low immunogenicity, they present a promising avenue for BAT engineering, offering a potentially more clinically translatable therapeutic approach.^36^ Therefore, further studies aimed at investigating the feasibility of engineering cells (such as ASCs) or adipose tissue to overexpress lncRNA AK029592 using both non-viral and viral methods are warranted. This approach could then involve isolating the sEVs that carry lncRNA AK029592 for potential therapeutic interventions in combating obesity and related metabolic disorders.

## Conclusion

lncRNA AK029592 was required for browning process of ASCs. Knocking down lncRNA AK029592 in BAT impaired CL316,243 mediated adipose tissue browning. Our findings revealed a novel lncRNA component of the thermogenic program that functioned as an endogenous miR-199a-5p sponge in beige adipocyte differentiation. These results might provide a novel therapeutic target and strategy for the future work in combating obesity and related metabolic diseases.

## Supplementary material

Supplementary material is available at *Stem Cells Translational Medicine* online.

szae056_suppl_Supplementary_Tables_S1_Figures_S1-S5

## Data Availability

All related lncRNA microarray data that support the findings of this study have been deposited at Gene Expression Omnibus with the accession number GSE196468. All data will be made available on request.
