# Supplementary material for: A novel long noncoding RNA AK029592 contributes to thermogenic adipocyte differentiation
Source: Stem Cells Transl Med. 2024 Aug 8;13(10):985–1000. doi: 10.1093/stcltm/szae056 (PMC11465168; doi:10.1093/stcltm/szae056)
Supplement: szae056_suppl_Supplementary_Tables_S1_Figures_S1-S5 [file szae056_suppl_supplementary_tables_s1_figures_s1-s5.pdf]

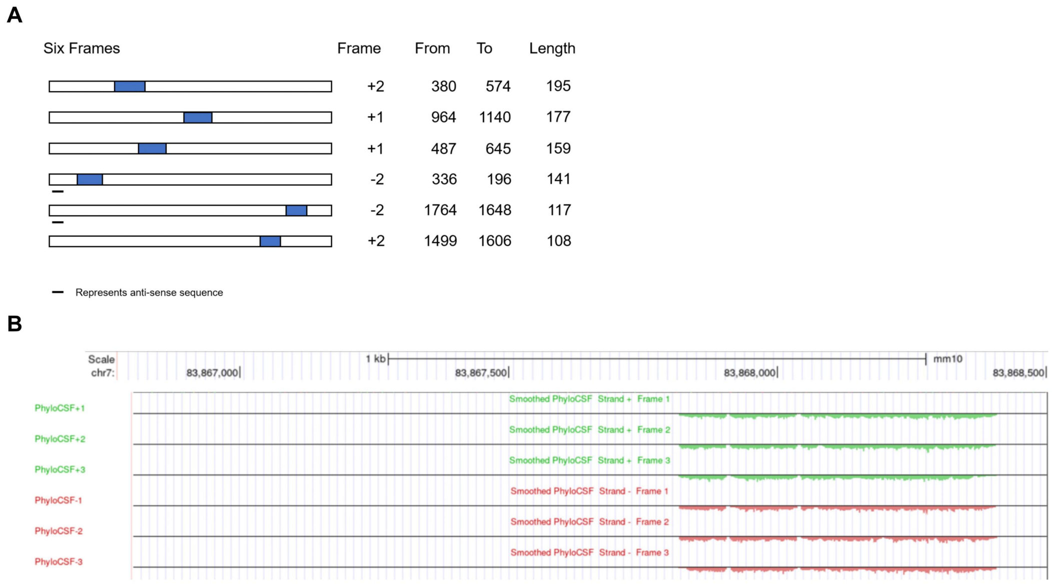

Supplementary Figure S1. The non-coding nature of lncRNA AK029592 was confirmed by coding-potential analysis. (A) Prediction of putative proteins encoded by lncRNA AK029592 using ORF Finder. (B) A screenshot of the PhyloCSF tracks for the validated lncRNA AK029592 shows a lack of sequence conservation throughout its genomic locus. Note that the PhyloCSF score is negative throughout the entire gene (for all six tracks), indicating a lack of sequence conservation, which supports its proper annotation as a noncoding RNA.

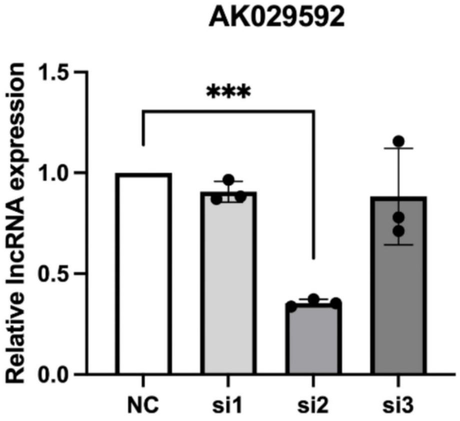

Supplementary Figure S2. The expression of lncRNA AK029592 could be suppressed by siAK20592. ASCs transfected with negative control or three independent siRNAs targeting lncRNA AK029592 were analysed by qPCR (n = 3). Data were represented as mean ± SD and analyzed by student t test, ***p<0.001.

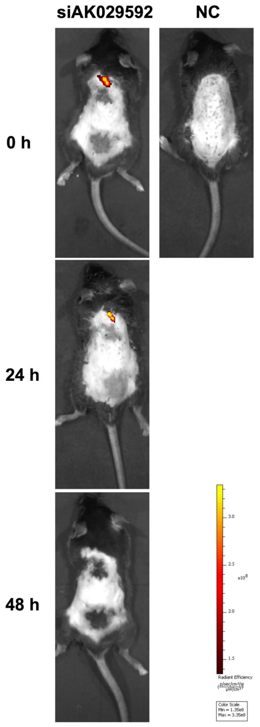

Supplementary Figure S3. The absorption of siAK029592 in BAT post-injection was monitored every 24h by *in vivo* bioluminescence imaging.

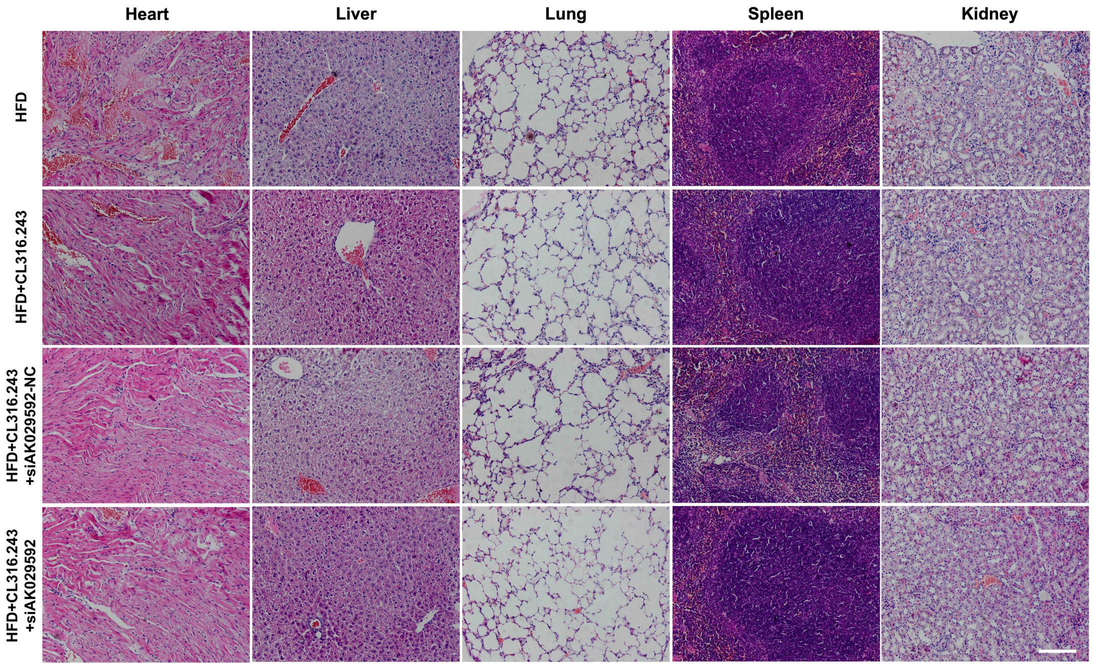

Supplementary Figure S4. Organ toxicity of lncRNA AK029592 *in vivo*. HE staining of each groups did not show obvious cardiac damage, pulmonary toxicity, spleen inflammatory infiltrates, liver and kidney injury. Scare bar = 100 μm.

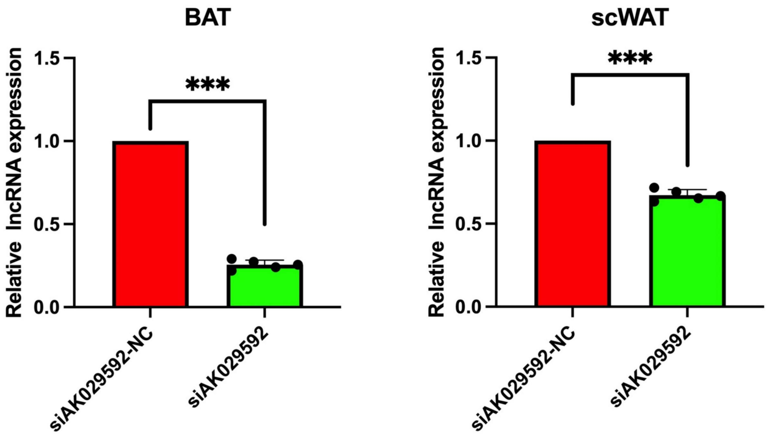

Supplementary Figure S5. The expression of lncRNA AK029592 in BAT and scWAT could be both suppressed by siAK20592 (n = 5). Data were represented as mean ± SD and analyzed by student t test, ***p<0.001.

Supplementary Table S1. Primers used for qPCR

Genes	Primers
Ucp1	5'- GGCATTCAGAGGCAAATCAGCT-3'
	CAATGAACACTGCCACACCTC
Pgc-1α	GGATATACTTTACGCAGGTCGA
	CGTCTGAGTTGGTATCTAGGTC
Cidea	CAATGTCAAAGCCACGATGTAC
	CTGTGCAGCATAGGACATAAAC
PPARγ	CCAAGAATACCAAAGTGCGATC
	TCACAAGCATGAACTCCATAGT
36B4	TGAGATTCGGGATATGCTGTTGG
	CGGGTCCTAGACCAGTGTTCT
AK029592	AGTCTGGGAACCATTAGCCTTAGGG
	ACTGTCAGCACAATAGCACCAACTC
mtND1	CCTATCACCCTTGCCATCAT
	GAGGCTGTTGCTTGTGTGAC
mtCox1	CTACTATTCGGAGCCTGAGC
	GCATGGGCAGTTACGATAAC
PECAM	ATGGAAAGCCTGCCATCATG
	TCCTTGTTGTTCAGCATCAC
miR-199a-5p	CCCAGTGTTCAGACTACCTGTTC
miR-1933-5p	CGCGCAGTCATGGTGTTCGGTCTTAGT
miR-743a-3p	GCCGGAAAGACACCAAGCTGAGTAGA
miR-490-5p	GGCCATGGATCTCCAGGTGGGT
miR-678	CCGTCTCGGTGCAAGGACTGGAG
miR-29a-3p	CCGCGCTAGCACCATCTGAAATCGGTT
miR-301a-3p	GCCGCGCAGTGCAATAGTATTGTCAAAGC
miR-297a-5p	GCGGGATGTATGTGTGCATGTGCATGT
miR-130a-3p	CCGCCAGTGCAATGTTAAAAGGGCAT
miR-9-3p	CCGCCGCATAAAGCTAGATAACCG
